# Dihydrotestosterone synthesis pathways from inactive androgen 5α-androstane-3β,17β-diol in prostate cancer cells: Inhibition of intratumoural 3β-hydroxysteroid dehydrogenase activities by abiraterone

**DOI:** 10.1038/srep32198

**Published:** 2016-08-26

**Authors:** Takashi Ando, Tsutomu Nishiyama, Itsuhiro Takizawa, Fumio Ishizaki, Yoshimichi Miyashiro, Keisuke Takeda, Noboru Hara, Yoshihiko Tomita

**Affiliations:** 1 Niigata University Graduate School of Medical and Dental Sciences, Department of Regenerative and Transplant Medicine, Niigata, 951-8510, Japan; 2 ASKA Pharmaceutical medical Co. Ltd., Kawasaki, 213-8522, Japan

## Abstract

Intratumoural dihydrotestosterone (DHT) synthesis could be an explanation for castration resistance in prostate cancer (PC). By using liquid chromatography-mass spectrometry, we evaluated the intratumoral DHT synthesis from 5α-androstane-3β,17β-diol (3β-diol), which is inactive androgen metabolized from DHT. 3β-diol had biochemical potential to be converted to DHT via three metabolic pathways and could stimulate PC cell growth. Especially, 3β-diol was not only converted back to upstream androgens such as dehydroepiandrosterone (DHEA) or Δ5-androstenediol but also converted directly to DHT which is the main pathway from 3β-diol to DHT. Abiraterone had a significant influence on the metabolism of DHEA, epiandrosterone and 3β-diol, by the inhibition of the intratumoural 3β-hydroxysteroid dehydrogenase (3β-HSD) activities which is one of key catalysts in androgen metabolic pathway. The direct-conversion of 3β-diol to DHT was catalysed by 3β-HSD and abiraterone could inhibit this activity of 3β-HSD. These results suggest that PC had a mechanism of intratumoural androgen metabolism to return inactive androgen to active androgen and intratumoural DHT synthesis from 3β-diol is important as one of the mechanisms of castration resistance in PC. Additionally, the inhibition of intratumoural 3β-HSD activity could be a new approach to castration-resistant prostate cancer treatment.

Prostate cancer (PC) is the most common cancer among men. In Japan, PC will be the leading cause of cancer morbidity in the near future and the mortality rate of PC in 2020 is anticipated to be 2.8-fold higher than it was in 2000^1^,^2^. Since *Huggins and Hodges* showed that surgical castration suppresses PC progression, it has been clear that androgen biosynthesis is important to the growth and survival of PC cells^3^.

Androgen deprivation therapy (ADT) has been the therapeutic mainstay for high-risk patients with metastatic PC, although the treatment effect is palliative in most cases. The majority of them have an initial response to ADT. However, most patients develop castration-resistant prostate cancer (CRPC), which is characterised by disease advancement with increasing levels of prostate-specific antigen (PSA) and/or deterioration of symptoms despite castration levels of plasma testosterone (T)^4^.

For the last several years, the importance of dehydroepiandrosterone (DHEA) biosynthesis in the adrenal gland has been an area of focus. Several studies have shown that intratumoural concentrations of T and dihydrotestosterone (DHT) are maintained and sufficiently activate androgen receptor (AR)-dependent transcriptomes in CRPC cells^5^,^6^,^7^,^8^. DHEA, the most common precursor of T and DHT in PC tissue during ADT^9^,^10^,^11^, is taken up by PC cells and converted to DHT in the cytoplasm and this metabolism called as adrenal-androgen-axis. This adrenal-androgen-axis is one of clarification of residual DHT in CRPC cells.

The androgens 5α-androstane-3α,17β-diol (3α-diol) and 5α-androstane-3β,17β-diol (3β-diol) are categorised as inactive androgens metabolised from DHT because they are unable to bind to the AR^12^,^13^,^14^ (Fig. 1). Moreover, several studies have shown that 3β-diol was reported to stimulate oestrogen receptor β (ERβ) and display antitumour effect in PC cell lines^15^,^16^,^17^,^18^,^19^. Although it is well known that 3α-diol and 3β-diol have no direct stimulation to AR, they have theoretically biochemical potential to be converted to most potent androgen, DHT, via androsterone (AND), or epiandrosterone (EpiAND) and androstanedione (5α-A-dione). Recently, *Chang et al.* reported the novel DHT synthesis pathway from 3α-diol via AND that has been called the back-door pathway^20^,^21^,^22^,^23^,^24^,^25^. Our laboratory also reported the other reformation pathway from 3β-diol to DHT via DHEA^26^. These studies suggest the existence of multiple DHT synthesis pathways from inactive androgens and could be a part of the mechanism behind castration resistance in PC.

The enzyme 3β-hydroxysteroid dehydrogenase (3β-HSD) is a key catalyst in androgen metabolism, converting DHEA to androstenedione (A-dione), Δ5-androstenediol (Δ5-Adiol) to T, EpiAND to 5α-A-dione and DHT to 3β-diol^27^. Few reports showed the existence of the direct-conversion from 3β-diol to DHT catalysed by 3β-HSD in mouse prostate, human adrenal grand and placenta^28^,^29^,^30^,^31^,^32^. There are two subtypes of 3β-HSD in humans. Type 1 (3β-HSD-1) of 3β-HSD is mainly expressed in prostate tissue, including PC and some malignant tumours, and type 2 (3β-HSD-2) is expressed in the adrenal gland^28^,^33^,^34^,^35^. It has been shown that 3β-HSD-1 has higher activity than 3β-HSD-2 and the activity of 3β-HSD-1 is strongly associated with the intratumoural conversion of DHEA to A-dione in the castration environment^27^,^28^,^33^,^34^. Therefore, 3β-HSD-1 has an important role in intratumoural androgen synthesis in CRPC.

On the other hand, as a result of the clinical success of abiraterone, attention has been focused on the enzyme CYP17A1. CYP17A1, unlike 3β-HSD, is a cytochrome P450 enzyme and one of the key enzymes of sexual steroid production. CYP17A1, which catalyses both 17α-hydroxylase and 17.20-lyase reactions, is involved in androgen production, converting cholesterol to DHEA in the adrenal gland and testis. Inhibition of CYP17A1 leads to the suppression of all androgens. Ketoconazole, a non-steroidal imidazole antifungal agent with non-selective CYP17 inhibition activity, has been used to treat advanced metastatic PC. High-dose ketoconazole therapy (HDKT) had shown a PSA response and prevented clinical cancer progression by the inhibition CYP17 enzyme activities in the adrenal gland^36^,^37^,^38^. However, little survival benefit had been shown because HDKT also inhibits other important metabolic enzymes, which can lead to severe adverse events. Experience with HDKT demonstrates that ketoconazole can prevent PC cell growth *in vivo* and suggests that it prevents intratumoural androgen metabolism^39^,^40^,^41^. Abiraterone and orteronel are selective CYP17A1 inhibitors. Abiraterone is a potent, irreversible inhibitor of CYP17A1, whereas orteronel is a selective, non-steroidal inhibitor of 17,20-lyase^42^. Selective inhibition improves their efficacies and toxicity profile, compared with non-selective, multi-activity CYP17 inhibition. Among the anticipated efficacies of these CYP17A1 inhibitors is the suppression of DHEA production in the adrenal gland. Additionally, *Li et al.* has shown that abiraterone can prevent the 3β-HSD-mediated conversion of DHEA to A-dione in PC cell lines *in vivo*^43^. This activity of abiraterone may be one of reasons why it had a stronger potential to suppress CRPC growth than the other two agents *in vivo*^44^,^45^. The ability of abiraterone to inhibit the activity of 3β-HSD in other intratumoural androgen pathways, and the abilities of ketoconazole and orteronel to inhibit the activity of 3β-HSD have not been studied.

In this study, we performed experiments designed to demonstrate intratumoural DHT synthesis pathways form 3β-diol in PC cells and to show the effect of CYP17 inhibitors on intratumoural androgen metabolism by 3β-HSD.

## Results

### Androgenic activities and the existence of back-conversion of 3β-diol or EpiAND to upstream androgens in the pathway of DHT synthesis in PC cells

**Figure 2:**
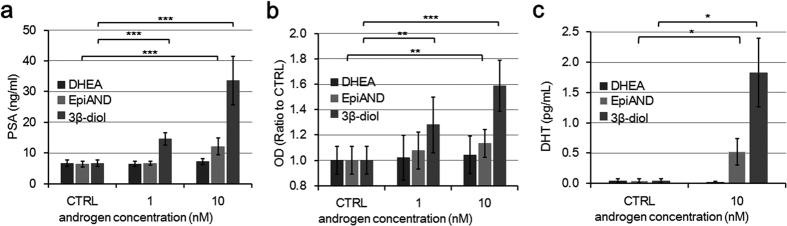
Androgenic activities of DHEA, EpiAND or 3β-diol. (**a**) PSA secretions into the medium by LNCaP cells (1 × 10^5^ cells/ml) treated with DHEA, EpiAND or 3β-diol (0, 1 or 10 nM) for 3 days. (**b**) Absorbance of MTS assay of LNCaP cells (1 × 10^5^ cells/ml) treated with DHEA, EpiAND or 3β-diol (0, 1 or 10 nM) for 3 days. Optical density (OD). (**c**) DHT levels secreted into the medium in LNCaP cells (1 × 10^5^ cells/ml) treated with 10 nM of DHEA, EpiAND or 3β-diol for 3 days were measured using LC-MS. PSA secretions and absorbances increased in the presence of EpiAND and 3β-diol in a concentration-dependent manner. At concentrations of 1 and 10 nM, 3β-diol exhibited higher androgenic activities than EpiAND in LNCaP cells. DHT levels were also higher in the presence of 3β-diol than in the presence of EpiAND. DHEA at 10 nM slightly stimulated LNCaP cell proliferation, but the stimulation was not significant and increase of DHT level was not measured. Data displayed are the mean ± s.d and are representative of at least three independent experiments. *P < 0.05, **P < 0.01, ***P < 0.001, vs. CTRL, by using t-test.

In order to assess androgenic activities of 3β-diol, DHEA and EpiAND, we measured PSA secretions, absorbance of MTS assay and changes of DHT levels secreted into the medium by LNCaP cells by using liquid chromatography-mass spectrometry (LC-MS) (Fig. 2). In the presence of each androgen, PSA secretions and absorbance were increased in a concentration-dependent manner. At a concentration of 10 nM, in particular, 3β-diol stimulated LNCaP cells stronger than DHEA or EpiAND. PSA secretions in the presence of 10 nM of DHEA, EpiAND and 3β-diol were 7.4 ± 0.9, 12.1 ± 2.7 and 33.6 ± 7.9 ng/ml (control, 6.7 ± 1.0 ng/ml), respectively, and EpiAND and 3β-diol stimulated PSA secretions significantly (both *P* < 0.001) (Fig. 2a). The ratios of absorbance in the presence of 10 nM DHEA, EpiAND and 3β-diol to that of the control (1.00 ± 0.11) were 1.04 ± 0.15, 1.13 ± 0.11 and 1.59 ± 0.20, respectively, and EpiAND and 3β-diol stimulated cell proliferations significantly (*P* < 0.01 and *P* < 0.01, respectively) (Fig. 2b). DHT levels in the presence of 10 nM of DHEA, EpiAND and 3β-diol were 0.02 ± 0.02, 0.52 ± 0.22 and 1.83 ± 0.57 pg/ml (control, 0.04 ± 0.03 pg/ml), respectively, and DHT levels were increased significantly by the addition of EpiAND and 3β-diol (both *P* < 0.05) (Fig. 2c). There were strong associations among PSA secretions, DHT and 3β-diol levels. The coefficient of correlation between PSA secretions and DHT levels, PSA secretions and 3β-diol levels, and DHT levels and 3β-diol levels were 0.83, 0.90 and 0.99, respectively (see Supplementary Fig. S1).

**Figure 3:**
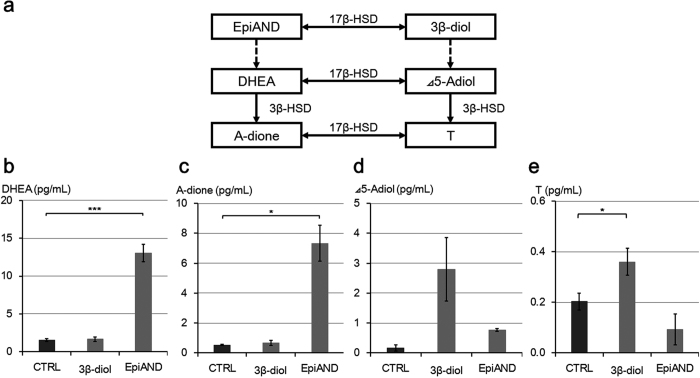
Back-conversion of 3β-diol or EpiAND to upstream androgen, Δ5-Adiol or DHEA, respectively. (**a**) Intratumoral androgen synthesis pathways of back-conversion. DHEA (**b**), A-dione (**c**), Δ5-Adiol (**d**) and T (**e**) levels secreted into the media by LNCaP cells (1 × 10^5^ cells/ml) treated with 10 nM of 3β-diol or EpiAND for 3 days were measured using LC-MS. By the addition of 3β-diol, DHEA, A-dione and Δ5-Adiol levels were not changed significantly (*P* = 0.64, *P* = 0.35 and *P* = 0.07, respectively), but T level was increased significantly (*P* = 0.03). By the addition of EpiAND, Δ5-Adiol and T levels were not changed significantly (*P* = 0.125 and *P* = 0.09, respectively), but DHEA and A-dione levels were increased significantly (*P* < 0.001 and *P* = 0.02, respectively). Data displayed are the mean ± s.d and are representative of at least three independent experiments. *P < 0.05, **P < 0.01, ***P < 0.001, vs. CTRL, by using t-test.

In order to assess back-conversion in detail, we measured levels of DHEA, A-dione, Δ5-Adiol and T in the medium in LNCaP cells treated with 10 nM of 3β-diol or EpiAND for 3 days using LC-MS (Fig. 3). We showed the scheme of back-conversion in Fig. 3a. By the addition of 3β-diol, DHEA, A-dione and Δ5-Adiol levels were not changed significantly (*P* = 0.64, *P* = 0.35 and *P* = 0.07, respectively), but T level was increased significantly (*P* = 0.03) (Fig. 3b–e). By the addition of EpiAND, Δ5-Adiol and T levels were not changed significantly (*P* = 0.125 and *P* = 0.09, respectively), but DHEA and A-dione levels were increased significantly (*P* < 0.001 and *P* = 0.02, respectively) (Fig. 3b–e). By the addition of EpiAND, DHEA and A-dione levels were increased higher than by the addition of 3β-diol (Fig. 3b,c). By the addition of 3β-diol, Δ5-Adiol and T levels were increased higher than in the presence of EpiAND (Fig. 3d,e). These results suggested that 3β-diol and EpiAND tended to be converted back to upstream androgens, Δ5-Adiol and DHEA, respectively.

### Inhibition of intraprostatic 3β-HSD mediated conversion by abiraterone

**Figure 4:**
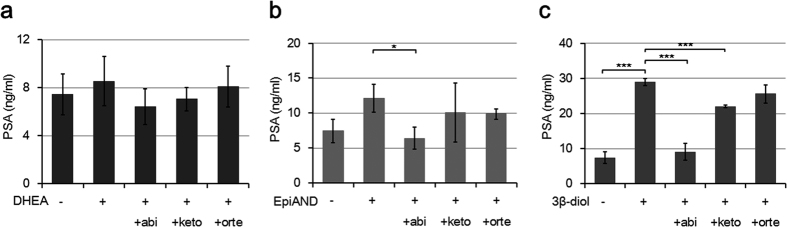
The influences of CYP17 inhibitors on PSA secretions in the presence of androgens. PSA secretions into the medium by LNCaP cells (1 × 10^5^ cells/ml) treated with 10 μM of each CYP17 inhibitor in the presence of 10 nM of DHEA (**a**), EpiAND (**b**) or 3β-diol (**c**) for 3 days. Abiraterone (abi) suppressed PSA secretions in the presence of EpiAND and 3β-diol (*P* = 0.033 and *P* < 0.001, respectively). Ketoconazole (keto) suppressed PSA secretions only in the presence of 3β-diol (*P* < 0.001) and orteronel (orte) did not suppress PSA secretions. Data displayed are the mean ± s.d and are representative of at least three independent experiments. *P < 0.05, **P < 0.01, ***P < 0.001, -androgen (CTRL) vs. +androgen, +androgen vs. +androgen +CYP17 inhibitor, by using t-test.

We checked the cytotoxicity of the CYP17 inhibitors abiraterone, ketoconazole and orteronel. These three agents did not show severe cell toxicity at a concentration of 10 μM (see Supplementary Fig. S2). PSA secretions into the medium by LNCaP cells in the presence of 10 nM of DHEA, EpiAND and 3β-diol were suppressed by the addition of each CYP17 inhibitor (Fig. 4a–c). Abiraterone suppressed PSA secretions in the presence of EpiAND and 3β-diol (*P* = 0.03 and *P* < 0.0001, respectively). Ketoconazole suppressed PSA secretions only in the presence of 3β-diol (*P* < 0.001) and orteronel did not suppress PSA secretions. These results suggested that abiraterone had the strongest influence on intratumoural androgen metabolism in CTP17 inhibitors.

**Figure 5:**
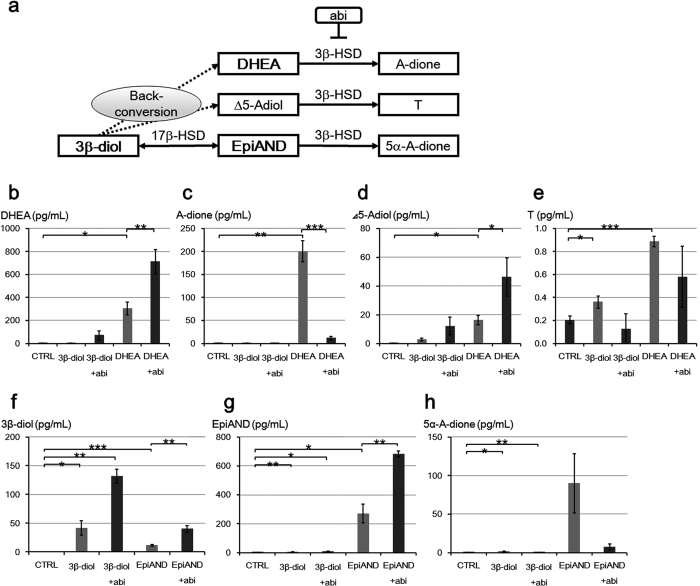
Abiraterone inhibited androgen metabolic pathways by the inhibition of 3β-HSD activities. (**a**) Androgen metabolic pathway catalysed by 3β-HSD and back-conversion from 3β-diol. Abiraterone (abi). DHEA (**b**), A-dione (**c**), Δ5-Adiol (**d**), and T (**e**) levels secreted into the medium in the presence of 10 nM of DHEA or 3β-diol by LNCaP cells (1 × 10^5^ cells/ml) treated with or without 10 μM abi for 3 days were measured using LC-MS. In the presence of 10 nM of EpiAND or 3β-diol, 3β-diol (**f**), EpiAND (**g**) and 5α-A-dione (**h**) levels secreted into the medium by LNCaP cells (1 × 10^5^ cells/ml) treated with or without 10 μM abi for 3 days were measured using LC-MS. DHEA and Δ5-Adiol levels were further increased by the addition of abi in the presence of DHEA (*P* < 0.01 and *P* < 0.05, respectively) and there were similar tendencies in the presence of 3β-diol (DHEA level; *P* = 0.125, and Δ5-Adiol level; *P* = 0.100) (**b**,**d**). The increases of A-dione and T levels were suppressed by the addition of abi in the presence of DHEA (*P* < 0.001 and *P* = 0.080, respectively) and there were similar tendencies in the presence of 3β-diol (A-dione level; *P* = 0.643 and T level; *P* = 0.242, respectively) (**c**,**e**). 3β-diol and EpiAND levels were further increased by the addition of abi in the presence of 3β-diol or EpiAND (3β-diol levels; *P* < 0.01 or *P* < 0.01, EpiAND levels; *P* < 0.05 or *P* < 0.01, respectively) (**f**,**g**). The increase of 5α-A-dione level was suppressed by the addition of abi in the presence of 3β-diol or EpiAND (*P* < 0.01 or *P* = 0.093, respectively) (**h**). Data displayed are the mean ± s.d and are representative of at least three independent experiments. *P < 0.05, **P < 0.01, ***P < 0.001, CTRL vs. +androgen, +androgen vs. +androgen +abi, by using t-test.

In order to assess whether abiraterone could inhibit the 3β-HSD activity or not, we measured androgen secretions into the medium by LNCaP cells treated with or without abiraterone 10 μM in the presence of 3β-diol, DHEA or EpiAND for 3 days using LC-MS (Fig. 5). We showed the schema of the metabolic pathway catalysed 3β-HSD including back-conversion and the inhibition of abiraterone in Fig. 5a. DHEA and Δ5-Adiol levels were further increased by the addition of abiraterone in the presence of DHEA (*P* < 0.01 and *P* < 0.05, respectively) and there were similar tendencies in the presence of 3β-diol (DHEA level; *P* = 0.125, and Δ5-Adiol level; *P* = 0.100) (Fig. 5b,d). The increases of A-dione and T levels were suppressed by the addition of abiraterone in the presence of DHEA (*P* < 0.001 and *P* = 0.080, respectively) and there were similar tendencies in the presence of 3β-diol (A-dione level; *P* = 0.643 and T level; *P* = 0.242, respectively) (Fig. 5c,e). 3β-diol and EpiAND levels were further increased by the addition of abiraterone in the presence of 3β-diol or EpiAND (3β-diol levels; *P* < 0.01 or *P* < 0.01, EpiAND levels; *P* < 0.05 or *P* < 0.01, respectively) (Fig. 5f,g). The increase of 5α-A-dione level was suppressed by the addition of abiraterone in the presence of 3β-diol or EpiAND (*P* < 0.01 or *P* = 0.093, respectively) (Fig. 5h). These results suggested that abiraterone could not inhibit back-conversion from 3β-diol, but abiraterone could inhibit the 3β-HSD activities in all three points demonstrated.

### DHT synthesis pathways from 3β-diol

**Figure 6:**
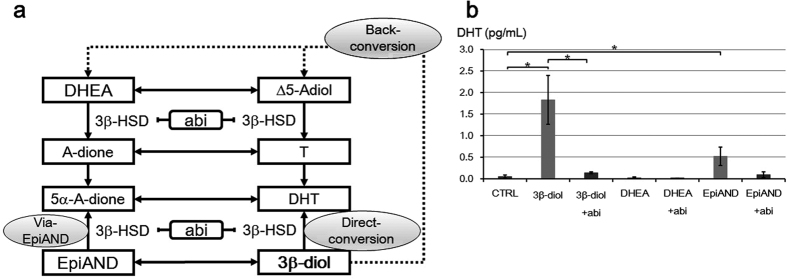
DHT synthesis pathways from 3β-diol. (**a**) Three metabolic pathway from 3β-diol to DHT including back-conversion, pathway via EpiAND and direct-conversion. (**b**) DHT levels secreted into the medium by LNCaP cells (1 × 10^5^ cells/ml) treated with or without 10 μM abiraterone (abi) in the presence of 10 nM of 3β-diol, DHEA or EpiAND for 3 days were measured using LC-MS. DHT level was increased in the presence of 3β-diol higher than in the presence of DHEA or EpiAND (*P* < 0.05, *P* = 0.421 or *P* < 0.05, respectively) (**b**). This increase of 3β-diol was suppressed by the addition of abi (*P* < 0.05). Data displayed are the means ± s.d. and are each representative of three independent experiments. *P < 0.05, **P < 0.01, ***P < 0.001, CTRL vs. +androgen, +androgen vs. +androgen +abi, by using t-test.

In order to assess main metabolic pathways of 3β-diol to DHT, we also measured DHT secretion into the medium by LNCaP cells treated with or without abiraterone 10 μM in the presence of 3β-diol, DHEA or EpiAND for 3 days using LC-MS (Fig. 6). We assumed three metabolic pathways from 3β-diol to DHT; via back-conversion, via EpiAND and 5α-A-dione, and direct-conversion catalysed 3β-HSD (Fig. 6a). In the presence of 3β-diol, DHT level was increased higher than in the presence of DHEA, however the levels of androgens as DHT resource such as DHEA, A-dione, Δ5-Adiol and T in the presence of 3β-diol were much lower than in the presence of DHEA (Figs 5b–e and 6b). Additionally, the increase of DHT in the presence of 3β-diol was significantly suppressed by the addition of abiraterone. These results suggested that main metabolic pathway of 3β-diol to DHT would not be via back-conversion and the main metabolic pathway should to be inhibited by abiraterone. On the other hand, in the presence of 3β-diol, DHT level was increased higher than in the presence of EpiAND, however 5α-A-dione level as DHT resource was much lower than in the presence of EpiAND (Figs 5h and 6b). These results suggested that the pathway from 3β-diol to DHT via 5α-A-dione was not the main metabolic pathway. Thus, the direct-conversion catalysed by 3β-HSD was the main metabolic pathway of 3β-diol to DHT and abiraterone could inhibit the intratumoural multiple DHT synthesis pathways from 3β-diol by blocking 3β-HSD activities.

## Discussion

In this study, we presented three important findings: the identification of intratumoural DHT synthesis pathways from 3β-diol including direct-conversion of 3β-diol to DHT; back conversion of 3β-diol to upstream androgens and inhibition of the intratumoural 3β-HSD-mediated conversion by abiraterone. The androgen 3β-diol is categorised as an inactive androgen because it cannot bind to the AR, and it may have an anti-prostate cancer potential by binding to ERβ^15^,^16^,^17^,^18^,^19^. In particular, DHT is converted to 3β-diol by 3β-HSD; however, in this study, 3β-diol was converted back to DHT and stimulated LNCaP cell growth. We confirmed three different metabolic pathways by which 3β-diol can be converted to DHT as shown in Fig. 6a. They are the known metabolic pathways via EpiAND and 5α-A-dione; back-conversion to DHEA and direct-conversion to DHT by 3β-HSD^26^,^27^,^28^,^29^,^30^,^31^,^32^. During ADT, DHEA produced in the adrenal gland is the main precursor of androgens for PC cells^9^,^10^,^11^. DHEA or Δ5-Adiol is metabolised to A-dione or T by 3β-HSD and A-dione or T is metabolised to 5α-A-dione or DHT by 5α-reductase. Because these activities of 3β-HSD and 5α-reductase proceed in only one direction, intratumoural androgen metabolism has been thought as a unidirectional pathway. However, as we showed in this study, 3β-diol is converted back to upstream androgens such as DHEA or Δ5-Adiol which was metabolised by 3β-HSD to A-dione or T, respectively. This back-conversion suggested the existence of a counter-flow in the intratumoural androgen cascade. We could not identify an enzyme working in this metabolic reversal, but the reversed flow was not inhibited by abiraterone, which suggests that the unknown enzyme may not be 3β-HSD.

We compared androgen profiles in the presence of DHEA, EpiAND, and 3β-diol at the same concentration. At concentrations of DHEA lower than physiological concentrations, DHEA was hardly metabolised to DHT via T. EpiAND was also hardly metabolised to DHT via 5α-A-dione. In the presence of 3β-diol, the level of DHT significantly increased, but levels of T or 5α-A-dione were lower than these levels in the presence of DHEA or EpiAND. Several reports showed the capacity of 3β-HSD to directly convert 3β-diol to DHT in the presence of NAD^+^ in human cells, adrenal grand and placenta^29^,^30^,^31^,^32^. This direct-conversion had been seen in mouse prostate cells, but there were no reports about the existence of the direct-conversion in human prostate cells. In this study, we affirmed the direct-conversion of 3β-diol to DHT using LC-MS, and this is the first report concerning direct-conversion in prostate cancer cells.

It had been suggested the existence of multiple mechanism of castration-resistance for PC. As one mechanism of CRPC progression, *Nishiyama et al.* had shown residual DHT in PC cells after receiving ADT was enough to stimulate PC cell growth^9^. The metabolism of 3β-diol including back-conversion and direct-conversion would be one reason of residual DHT. This phenomenon could be hardly applied to the mechanism of androgen-independent CRPC, which did not require androgens including DHT for its survival and growth, because our LC-MS results suggested that 3β-diol could not stimulate AR directly. In our real-time polymerase chain reaction (RT-PCR) result, AR related genes such as KLK3 and TMPRSS2 were increased by the addition of 3β-diol in the medium and abiraterone strongly suppressed these increases of mRNA (see Supplementary Fig. S3). These RT-PCR results supported our findings that 3β-diol could not stimulate AR directly and 3β-diol promoted LNCaP cells due to the conversion to DHT by 3β-HSD. LNCaP cells could survive and proliferate in the CS-FBC medium with 3β-diol, whether LNCaP cells could not grow in the medium without 3β-diol (see Supplementary Fig. S4). We also affirmed that 3β-diol could promote cell growth of VCaP, which expressed wild type AR, not only LNCaP cells, which expressed mutant AR and these cell lines required androgens for its survival (see Supplementary Fig. S5)^46^,^47^. Thus, our findings would be one approach for the resolution of the mechanism with the object of intratumoral androgen synthesis in CRPC, which had remaining androgen dependence.

Ketoconazole has traditionally been used to treat CRPC in America and Europe; abiraterone and orteronel are among the new therapeutic agents for CRPC patients. Some reports have suggested that ketoconazole prevents intratumoural androgen metabolism, but the effect had not been clear^39^,^40^,^41^. *Rue et al.* showed that abiraterone could inhibit the activity of the 3β-HSD-catalysed conversion of DHEA to A-dione^43^. In this study, we could not demonstrate the significant prevention of intratumoural androgen metabolism by ketoconazole or orteronel (data did not shown). However, abiraterone significantly inhibited the androgenic activity of DHEA, EpiAND and 3β-diol, thereby showing its potential to prevent the effects of intratumoural androgen metabolism.

By adding abiraterone in the presence of those each androgens, androgen profiles were significantly changed. These changes were found at same points of the androgen metabolism pathway catalysed by 3β-HSD^27^,^32^. 3β-HSD-1 is expressed in prostate tissue, including PC cells. *In vitro* studies have strongly associated the activity of 3β-HSD-1 with the intratumoural conversion of DHEA to A-dione in the castration environment^27^,^28^,^33^,^34^. We performed knockdown of 3β-HSD-1 by using small interfering RNA (siRNA) technique for the affirmation of importance of 3β-HSD-1. PSA secretions by LNCaP cells treated siRNA of 3β-HSD-1 were significantly decreased (see Supplementary Fig. S6). Additionally, we confirmed the expressions of several androgen metabolic enzyme mRNA using RT-PCR (see Supplementary Fig. S7). The gene expressions of 17β-hydroxysteroid dehydrogenase 6 (HSD17B6), 10 (HSD17B10), retinol dehydrogenase 16 (all-trans) (RDH16) and retinol dehydrogenase 5 (RDH5) were detected in higher level than the expression of HSD3B1, however those enzymes were known as catalysts as 3α-HSD^48^. Therefore, we concluded that 3β-HSD-1 was most important enzyme which convert 3β-diol to DHT especially via direct-conversion. This is the first report to demonstrate that abiraterone could inhibit 3β-HSD-1 activity in all four points in the intratumoural androgen metabolic pathway.

One group has conducted a trial of intratumoural inhibition by abiraterone in CRPC patients in which the abiraterone was taken with food because taking abiraterone with food increases its blood concentration several fold, compared with taking abiraterone without food^44^,^45^,^49^. However, this trial did not show significant improvement. There may be several reasons why it is hard to directly apply this intratumoural inhibition to CRPC patients. We thought the strongest factor was the concentration of abiraterone. Looking at our study, it was clear that abiraterone could inhibit intratumoral 3β-HSD-1, but to exert this inhibition in PC cells, we needed a concentration of abiraterone higher than that usually used to treat CRPC patients^44^,^45^. In our study, intratumoural inhibition of 3β-HSD-1 by abiraterone had occurred in a concentration-dependent manner in the presence of 1–10 μM abiraterone. However, at abiraterone concentrations lower than 1 μM, we could not find significant inhibition (data did not shown). When we treat CRPC patients by inhibiting the activity of CYP17A1 in the adrenal gland and suppressing DHEA production, abiraterone is used at blood levels near 1 μM. Therefore, to successfully apply this intratumoral inhibition strategy using abiraterone to CRPC patients, we need to find a way to increase and maintain the blood abiraterone level. We would also need to monitor the blood abiraterone level for safety purposes. The experience and knowledge described here suggests the importance of thinking about the intratumoural androgen environment and the possibility of inhibiting the intratumoural activity of 3β-HSD-1 as a new anti-prostate cancer treatment. Selective 3β-HSD-1 inhibitors would have stronger efficacy, selectivity for prostate cancer and less toxicity to the adrenal gland than abiraterone.

Recently, *Zhenfei et al.* reported a new association between abiraterone and 3β-HSD-1^50^. They reported that abiraterone had been converted to Δ4-abirateorne (Δ4-abi) by 3β-HSD-1. They also reported that Δ4-abi had several-fold stronger potential to inhibit the activities of 3β-HSD-1, CYP17A1, 5α-reductase and AR in PC cells than abiraterone. Interestingly, the overexpression of 3β-HSD-1 stimulated PC cell growth; however, in the presence of abiraterone, 3β-HSD-1 would help to suppress PC cell growth by converting abiraterone to Δ4-abi. These findings may represent another avenue for the discovery of new anti-prostate cancer agents. As described above, clarifying intratumoural androgen metabolism, including the function of 3β-HSD-1, would provide deeper insight into the mechanism of castration resistance in PC and identify possible avenues for the development of new CRPC treatments.

In conclusion, we affirmed that three metabolic pathways of 3β-diol to DHT, back-conversion to DHEA, via 5α-A-dione and direct-conversion by 3β-HSD, and these metabolism formed intratumoural DHT synthesis from inactive androgens. Abiraterone had the potential to inhibit intratumoral androgen metabolisms by the inhibition intratumoural 3β-HSD activities, not only to inhibit adrenal CYP17A1 activities. Intratumoural 3β-HSD activities, especially 3β-HSD-1 would be a new approach to treat CRPC.

## Methods

### Cell lines and drugs

Human prostate cancer cell line, LNCaP cells and VCaP cells were purchased from the American Type Culture Collection. DHEA and EpiAND were purchased from Sigma Chemicals (St Louis, MO, USA). The 3β-diol was purchased from Atomax Chemicals Co. (Shenzhen, China). Ketoconazole was purchased from Sigma Chemicals. Abiraterone was purchased from Ark Pharm Inc. (Libertyville, IL, USA). Orteronel was purchased from ChemScene LLC (Monmouth Junction, NJ, USA). Androgens and CYP17 inhibitors were dissolved in pure ethanol or dimethyl-sulfoxide (DMSO). Final concentrations of ethanol and DMSO did not exceed 0.2% for any of the test substances. The doses of these compounds used in this study do not affect cell proliferation or survival.

### Cell culture and drug treatment

Cells were cultured in RPMI 1640 (Gibco Invitrogen, Grand Island, NY, USA) supplemented with 10% heat-inactivated foetal bovine serum (FBS), 1% MEM nonessential amino acids, 1% MEM sodium pyruvate solution 100 mM, 0.14% NaHCO_3_, and 80 mg/L of kanamycin, at 37 °C in a humidified 5% CO_2_ atmosphere. Cells were grown to sub-confluence and switched to steroid hormone-depleted medium without phenol-red, containing 10% charcoal-dextran stripped FBS (Biowest, Paris, France), with various concentrations of pharmacological agents for 3 days. In all experiments, the concentration of LNCaP cells and VCaP cells were 1 × 10^5^ cells/ml and 2 × 10^5^ cells/ml, respectively. For Tandem-R-PSA tests (Beckman Coulter Inc., San Diego, CA, USA), cells were cultured in 25 cm^3^ cell culture flasks with 5 ml medium in triplicates. Experiments were performed with each androgen, in the presence or absence of each CYP17 inhibitor. Entire experiment was performed thrice. To determine the levels of androgens, including DHEA, Δ5A-diol, A-dione, T, DHT, 5α-A-dione, EpiAND and 3β-diol in the culture medium, cells were cultured in 75 cm^3^ cell culture flasks with 10 ml medium in the presence of 10 nM of each androgen, with or without 10 μM of CYP17 inhibitors for 3 days. After the measurement of PSA levels of culture medium, levels of androgens in the culture medium were determined using LC-MS, as described below.

### MTS assay

Cells were cultured in 96-well flat-bottom plates with 100 μl medium in the presence of agents for 3 days. At the setting point, 20 μl of MTS solution (CellTiter 96® AQueous One Solution Cell Proliferation Assay; Promega, Madison, WI, USA) was added to each well. According to manufacturer’s instructions, the absorbance, which indicated relative cell proliferation and was shown as Optical density (OD) in figures, was determined using 490 nm filter and an iMark microplate reader (Bio-Rad Laboratories, Inc.). Results under each set of conditions were determined in triplicate or quadruplicate wells. All experiments were performed thrice.

### LC-MS analysis

Levels of androgens, including DHEA, Δ5-Adiol, A-dione, T, DHT, 5α-A-dione, EpiAND and 3β-diol, in the cell culture medium were determined using LC-MS using the procedure described by *Takizawa. et al.*^51^. In brief, the culture medium was extracted with ethyl acetate, and then the extracts were purified using a solid-phase extraction cartridge. After derivatization to picolinate ester forms, the concentrations of steroids were determined using LC-MS. Androgen levels were indicated as ‘pg/ml’. The limits of quantification of DHEA, A-diol, A-dione, T, DHT, 5α-A-dione, EpiAND and 3β-diol were 2, 0.5, 0.5, 0.5, 0.5, 1, 0.5 and 1 pg/assay, respectively. All experiment were performed thrice.

### RNA extraction and RT-PCR

LNCaP cells were treated with 10 nM 3β-diol with or without 10 μM abi for 3 days. After incubation, total RNA was isolated from the cells using the RNAqueous®-4PCR Kit (Ambion, Austin, TX, USA), and cDNA was generated using the High-Capacity cDNA Reverse Transcription Kit® (Applied Biosystems, Foster City, CA, USA), according to the manufacturer’s instructions. Incubation conditions for the generation of cDNA were as follows: 10 min at 25 °C and 2 h at 37 °C. RT-PCR experiments were carried out using a standard TaqMan PCR protocol according to the manufacturer’s recommendations (Applied Biosystems). Transcript of the housekeeping gene, β-actin (ACTB), was measured as the internal control. Assays were carried out using the ABI 7500 Real Time PCR system, Taqman Gene Expression assay mix® (Applied Biosystems) and Taqman Gene Expression assay (gene name and assay ID were shown in Supplemental T1). PCRs were carried out after incubation at 50 °C for 2 min and denaturing at 95 °C for 10 min, followed by 40 cycles at 95 °C for 15 sec and 60 °C for 1 min. Quantification of target gene expression in samples was accomplished by measuring the fractional cycle number at which the amount of expression reached a fixed threshold (CT). Relative quantification was given by CT values, determined by triplicate reactions. Triplicate CT values were averaged and ACTB CT was subtracted to obtain ΔCT. ΔΔCT was then calculated by subtracting ΔCT of the control (cells incubated in CS-FBS medium without any drugs for 3 days) from ΔCT of the sample. Relative expression levels were determined as 2^−ΔΔCT^. All experiments were performed thrice.

### Cell proliferation

To assess sufficiency of 3β-diol for LNCaP cells survival, cumulative differences in proliferation were measured. LNCaP cells were plated 1 × 10^6^ cells per 75 cm^3^ flasks in 10 ml phenol-red free medium with 10% CS-FBS with or without 10 nM 3β-diol. The cell culture medium were changed every 3 days and cell numbers were counted every 3 days. For cell counts, trypan blue was used to measure cell viability. All data were reported as numbers of viable cells counted using a cell counter (Countess® II FL Automated Cell Counter, Invitrogen).

### Transfection by using electroporation

LNCaP cells in steroid hormone-depleted medium without phenol-red, containing 10% CS-FBS were prepared for transfection. One × 10^5^ LNCaP cells were re-suspended in 100 μl resuspension buffer R (Neon® Transfection System; Invitrogen, Carlsbad, CA, USA) with 2 μM siRNA for HSD3B1 (s6926; Silencer® select, Ambion) or control non-silencing siRNA (#1 siRNA; Ambion) and transfected in 100 μl Neon tip with Neon transfection system (Invitrogen) using two pulses (1250 V input pulse voltage/20 ms input pulse width). Five × 10^4^ transfected cells in 500 μl phenol-red free medium containing 10% CS-FBS with 10 nM 3β-diol were plated each well of 24 well plate in triplicate and cultured for 3 days before Tandem-R PSA tests.

### Data analysis and Statistical methods

Differences between experimental data were analysed by Student’s t test. Pearson’s correlation coefficients were used to assess the relationship between experimental data. All analyses were performed using SPSS version15.0J (SPSS IN., Chicago, IL, USA) on a Windows-based computer.

## Additional Information

**How to cite this article**: Ando, T. *et al.* Dihydrotestosterone synthesis pathways from inactive androgen 5α-androstane-3β,17β-diol in prostate cancer cells: Inhibition of intratumoural 3β-hydroxysteroid dehydrogenase activities by abiraterone. *Sci. Rep.* **6**, 32198; doi: 10.1038/srep32198 (2016).

## Supplementary Material

Supplementary Information

## Figures and Tables

**Figure 1:**
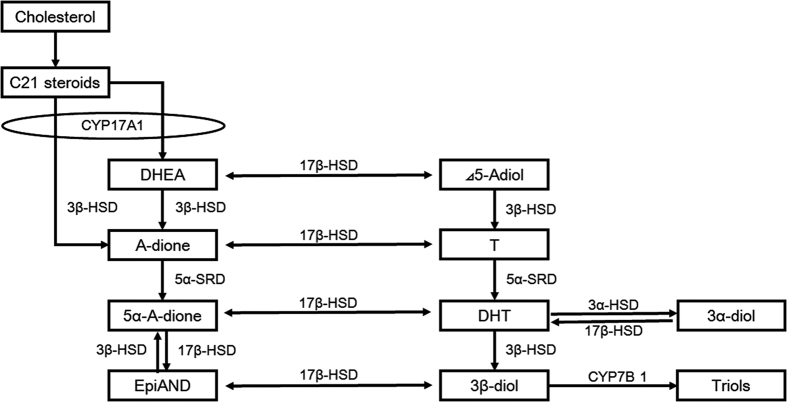
Intratumoural androgen metabolism in prostate cancer cells. C21 steroids (pregnenolone and progesterone) are converted to androgens, such as dehydroepiandrosterone (DHEA) and androstenedione (A-dione) by the sequential hydroxylase and lyase activities of CYP17A1 in the adrenal gland. Enzymes such as 3β-hydroxysteroid dehydrogenase (3β-HSD), 17β-hydroxysteroid dehydrogenase (17β-HSD) and steroid 5α-reductase (5α-SRD) participate in the intratumoural androgen metabolic pathway. The enzyme 3β-HSD metabolises DHEA to A-dione, Δ5-androstenediol (Δ5-Adiol) to testosterone (T) and epiandrosterone (EpiAND) to androstanedione (5α-A-dione). Dihydrotestosterone (DHT) is reduced to 5α-androstane-3α, 17β -diol (3α-diol) and 5α-androstane-3β, 17β-diol (3β-diol) by 3α-hydroxysteroid dehydrogenase (3α-HSD) and 3β-HSD, respectively. 3β-diol is hydroxylated by cytochrome P450-7B1 (CYP7B1) to triols.
