# Supplementary material for: Dihydrotestosterone synthesis pathways from inactive androgen 5α-androstane-3β,17β-diol in prostate cancer cells: Inhibition of intratumoural 3β-hydroxysteroid dehydrogenase activities by abiraterone
Source: Sci Rep. 2016 Aug 26;6:32198. doi: 10.1038/srep32198 (PMC4999866; doi:10.1038/srep32198)
Supplement: Supplementary Information [file srep32198-s1.pdf]

Dihydrotestosterone synthesis pathways from inactive androgen 5α-androstane-3β,17β-diol in prostate cancer cells: Inhibition of intratumoural 3β-hydroxysteroid dehydrogenase activities by abiraterone

Takashi Ando[1], Tsutomu Nishiyama[1,*], Itsuhiro Takizawa[1], Fumio Ishizaki[1], Yoshimichi Miyashiro[2], Keisuke Takeda[1], Noboru Hara[1], Yoshihiko Tomita[1]

[1]Niigata University Graduate School of Medical and Dental Sciences, Department of Regenerative and Transplant Medicine, Niigata, 951-8510, Japan

[2]ASKA Pharmaceutical medical Co. Ltd., Kawasaki, 213-8522, Japan

[*]nisiyama@med.niigata-u.ac.jp

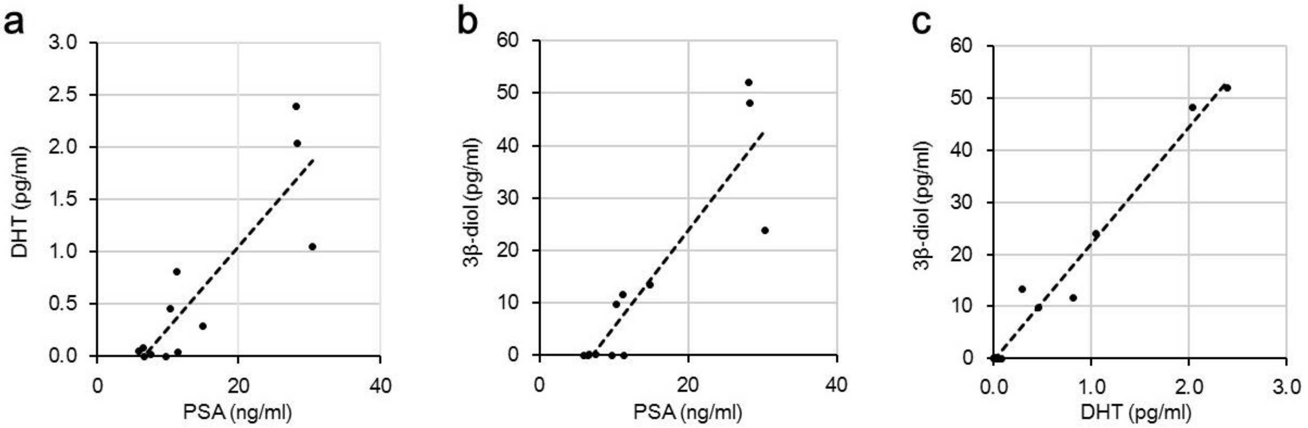

Figure S1. Associations among PSA secretions, DHT levels and 3β-diol levels in the medium. (A) Association between PSA secretions and DHT levels in the medium. Coefficient of correlation (r^2) = 0.83. (B) Association between PSA secretions and 3β-diol levels in the medium; r^2 = 0.90. (C) Association between DHT levels and 3β-diol levels in the medium; r^2 = 0.99.

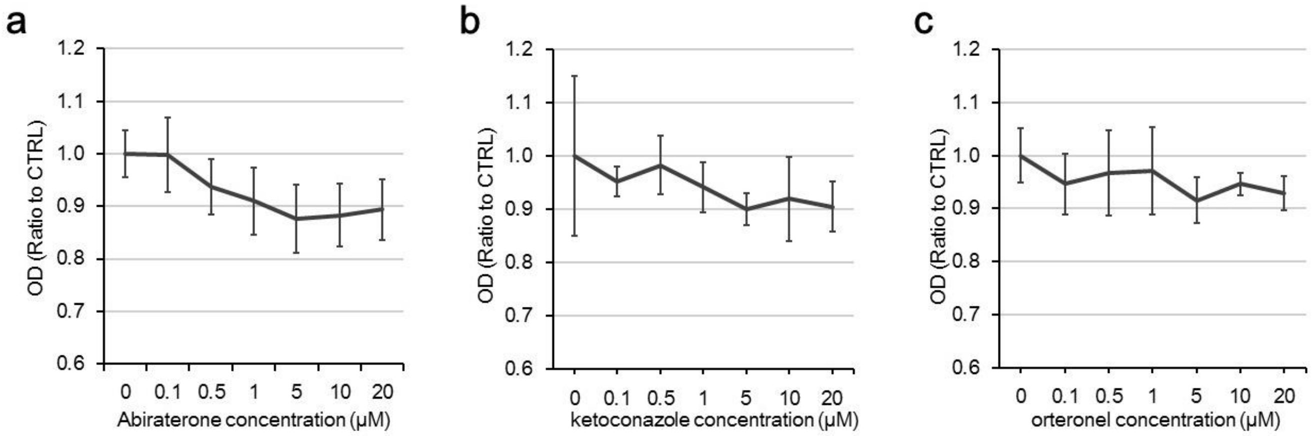

Figure S2. Cellular toxicities of CYP17 inhibitors. (A) Absorbances [optical density (OD)] of MTS assay of LNCaP cells (1×10^5 cells/ml) treated with various concentrations of abiraterone (A), ketoconazole (B) and orteronel (C) for 3 days. OD decreased in the presence of 10 μM abiraterone, ketoconazole or orteronel, compared with CTRL (OD ratio to CTRL, 0.88 ± 0.06, 0.92 ± 0.08, 0.95 ± 0.02, respectively) OD did not decrease when CYP17 inhibitors were added at concentrations lower than 0.1 μM (data did not shown). Data displayed are the means ± s.d. and are each representative of at least three independent experiments.

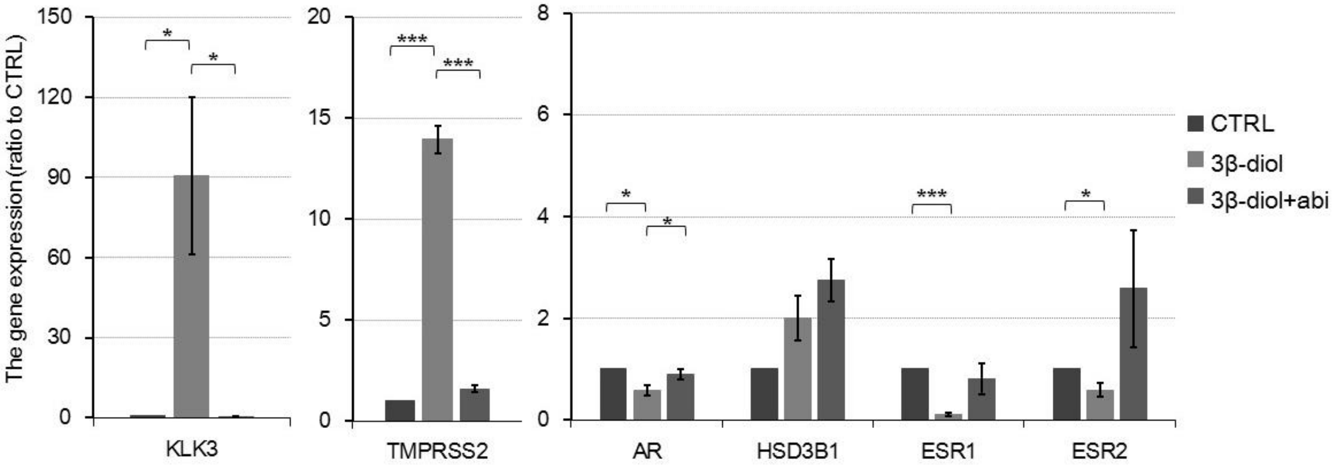

Figure S3. The gene expressions in LNCaP cells treated with 3β-diol and abiraterone. The gene expressions in LNCaP cells treated with or without 10 μM abiraterone (abi) in the presence or absence of 10 nM 3β-diol were measured by qRT-PCR. Kallikrein related peptidase 3 (KLK3), transmembrane protease serine 2 protein (TMPRSS2), androgen receptor (AR), 3β-hydroxysteroid dehydrogenase type 1 (HSD3B1), estrogen receptor 1 (ESR1) and estrogen receptor 2 (ESR2). In the presence of 3β-diol, the expressions of KLK3 and TMPRSS2 were increased and the expressions of AR, ESR1 and ESR2 were decreased. By the addition of abi, the expressions of KLK3 and TMPRSS2 were suppressed and the expression of AR was increased compared with those without abi. Data displayed are the mean ± s.d and are representative of three independent experiments. *$P < 0.05$, ***$P < 0.001$, CTRL vs. 3β-diol, 3β-diol vs. 3β-diol +abi, by using t-test.

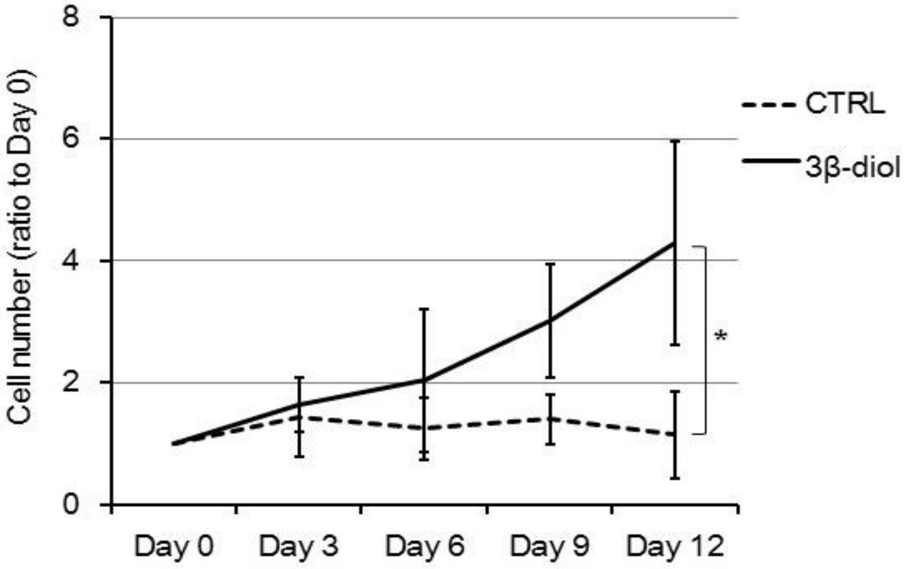

Figure S4. Proliferations of LNCaP cells in the presence of 3β-diol in the medium.

The numbers of LNCaP cells, which were incubated with or without 10 nM 3β-diol, were counted by every three days. LNCaP cells could continually proliferate in the presence of 10 nM 3β-diol ($P = 0.024$). Data displayed are the mean ± s.d and are representative of three independent experiments. *$P < 0.05$, CTRL vs. 3β-diol in each counted day, by using t-test.

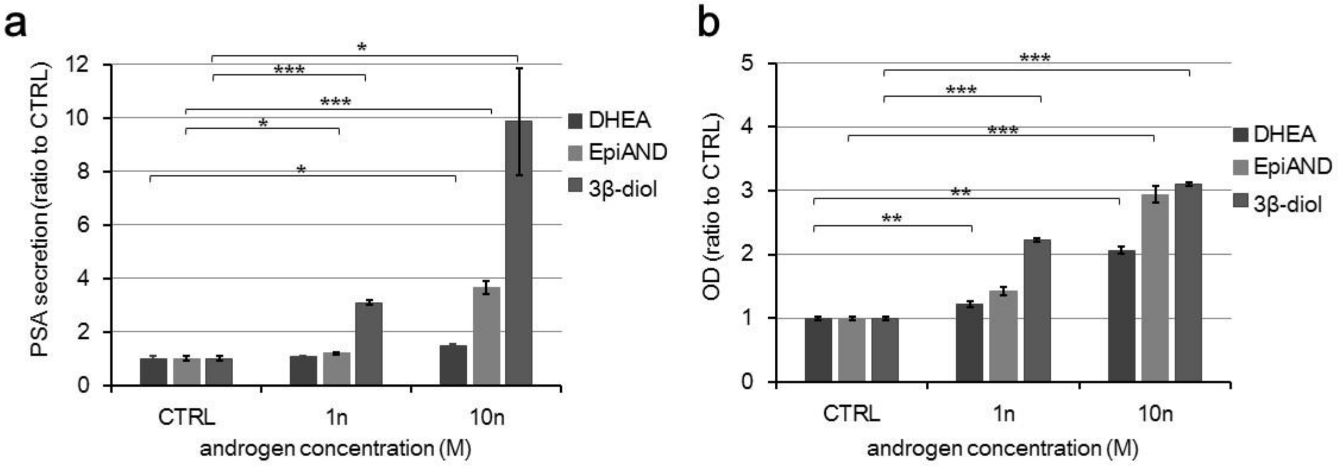

Figure S5. Androgenic activities of DHEA, EpiAND or 3β-diol for VCaP cells. (A) PSA secretions into the medium by VCaP cells (2×10^5 cells/ml) treated with DHEA, EpiAND or 3β-diol (0, 1 or 10 nM) for 3 days. (B) Absorbance of MTS assay of LNCaP cells (2×10^5 cells/ml) treated with DHEA, EpiAND or 3β-diol (0, 1 or 10 nM) for 3 days. Optical density (OD). PSA secretions and absorbances increased in the presence of each androgens in a concentration-dependent manner. At concentrations of 10 nM, every androgens could stimulate both cell proliferation and PSA secretion of VCaP. Data displayed are the mean ± s.d and are representative of three independent experiments. $*P < 0.05$, $**P < 0.01$, $***P < 0.001$, vs. CTRL, by using t-test.

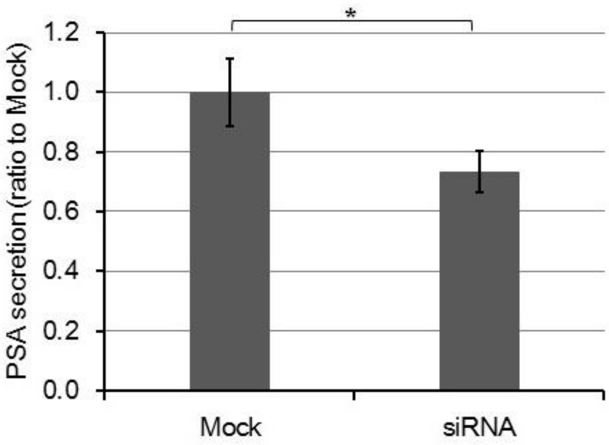

Figure S6. Reduction of androgenic activity of 3β-diol for LNCaP cells by siRNA of HSD3B1. PSA secretion into the medium by LNCaP cells transfected siRNA of control or HSD3B1 in the presence of 10 nM 3β-diol. PSA secretions by LNCaP cells transfected siRNA of HSD3B1 was reduced significantly (P = 0.013). Data displayed are the mean ± s.d and are representative of four independent experiments. *P < 0.05, Mock vs. siRNA, by using t-test.

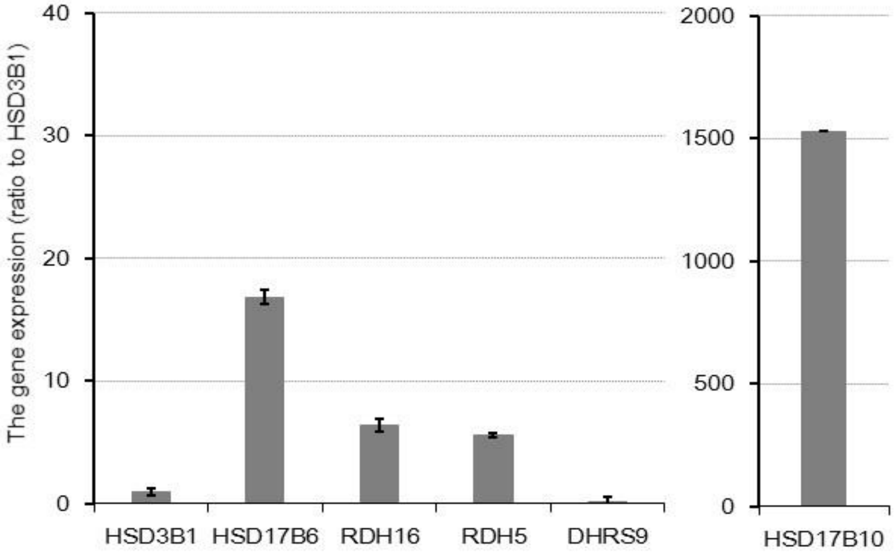

Figure S7. The gene expressions of androgen metabolic enzymes in LNCaP cells treated with 3β-diol. The gene expressions in LNCaP cells treated with 10 nM 3β-diol were measured by using qRT-PCR. 17β-hydroxysteroid dehydrogenase 6 (HSD17B6), 10 (HSD17B10), retinol dehydrogenase 16 (all-trans) (RDH16), retinol dehydrogenase 5 (RDH5) and short-chain dehydrogenase/reductase member 9 (DHRS9). The expressions of HSD17B6, HSD17B10, RDH16 and RDH5 were detected in higher level than the expression of HSD3B1. Data displayed are the mean ± s.d and are representative of three independent experiments.

Gene name	Assay ID
ACTB	Hs99999903_m1
AR	Hs00171172_m1
DHRS9	Hs00608375_m1
ESR1	Hs00174860_m1
ESR2	Hs00230957_m1
HSD3B1	Hs00426435_m1
HSD17B6	Hs00366258_m1
HSD17B10	Hs00189576_m1
KLK3	Hs02576345_m1
RDH5	Hs00161263_m1
RDH16	Hs00559712_m1
TMPRSS2	Hs00237175_m1

Table S1. Primers for qRT-PCR. Gene name and Taqman Gene Expression assay ID were shown in the table.
